# Quantitative Analysis of Axonal Branch Dynamics in the Developing Nervous System

**DOI:** 10.1371/journal.pcbi.1004813

**Published:** 2016-03-21

**Authors:** Kelsey Chalmers, Elizabeth M. Kita, Ethan K. Scott, Geoffrey J. Goodhill

**Affiliations:** 1 Queensland Brain Institute, The University of Queensland, Brisbane, Queensland, Australia; 2 School of Biomedical Sciences, The University of Queensland, Brisbane, Queensland, Australia; 3 School of Mathematics and Physics, The University of Queensland, Brisbane, Queensland, Australia; Imperial College London, UNITED KINGDOM

## Abstract

Branching is an important mechanism by which axons navigate to their targets during neural development. For instance, in the developing zebrafish retinotectal system, selective branching plays a critical role during both initial pathfinding and subsequent arborisation once the target zone has been reached. Here we show how quantitative methods can help extract new information from time-lapse imaging about the nature of the underlying branch dynamics. First, we introduce Dynamic Time Warping to this domain as a method for automatically matching branches between frames, replacing the effort required for manual matching. Second, we model branch dynamics as a birth-death process, i.e. a special case of a continuous-time Markov process. This reveals that the birth rate for branches from zebrafish retinotectal axons, as they navigate across the tectum, increased over time. We observed no significant change in the death rate for branches over this time period. However, blocking neuronal activity with TTX slightly increased the death rate, without a detectable change in the birth rate. Third, we show how the extraction of these rates allows computational simulations of branch dynamics whose statistics closely match the data. Together these results reveal new aspects of the biology of retinotectal pathfinding, and introduce computational techniques which are applicable to the study of axon branching more generally.

## Author Summary

The complex morphologies of neurons present challenges for analysis. Large data sets can be gathered, but extracting meaningful data from the hundreds of branches from one axon over a few hundred time points can be difficult. One problem in particular is matching a single unique branch through several images, when the branches can extend, retract, or be removed entirely. In addition, if the imaging is done in vivo, the environment itself can grow and shift. Here we introduce Dynamic Time Warping (DTW) analysis to follow the complex structures of neurons through time. DTW identifies individual branches and therefore allows the determination of branch lifetimes. Using this approach we find that for retinal ganglion cell axons, the branch birth rate increases over time as axons navigate to their targets, and that blocking neural activity slightly increases the branch death rate without impacting the birth rate. From the estimated birth and death rate parameters we create simulations based on a continuous-time Markov chain process. These tools expand the techniques available to study the development of neuronal structures and provide more information from large time-lapse imaging datasets.

## Introduction

A major question in neuroscience is how the early wiring of the brain is generated. Neurons extend axons and dendrites both locally and over long distances to create complex patterns of connections [1, 2]. The early connections must maintain a balance of precision and adaptability in their targeting [3]. Patients with developmental disorders, including autism and Tourette’s syndrome, show alterations in brain wiring [4, 5] and disruptions in axon guidance genes [6]. Late onset neurological conditions including Huntington’s, Parkinson’s and schizophrenia, can also be linked to changes in brain connectivity [7, 8] and axon guidance defects [9–12]. An understanding of how the wiring initially develops in normal and altered conditions is thus critical to understanding both neurodegeneration and potential treatment options.

The morphology of early axons is an important basis for their later function. Axons and dendrites often branch as they search for connecting partners [13–20]. Transitive, exploratory branching can also be a dynamic way to search a large swath of the environment en route to the target in order to read a wide variety of navigational cues [15]. Branching can also be focused in a small region in order to increase the number and density of synaptic contacts between cells. In both instances, these patterns of branches are dynamic and change with time.

Accordingly, the quantitative analysis of these complex branching patterns can be challenging. Between two static images taken minutes apart, branches can be born, extend, retract or die, as well as shift in space, since the surrounding environment also grows. Significant changes in the axon morphology must be separated from background noise in the image. Massive quantities of time-lapse data can be generated from developmental studies of cell morphology and connectivity [21–23]. Merely counting changes in the total number of branches between each time point [21, 23, 27–30] does not capture important aspects of branching, such as branch lifetimes. To measure these aspects requires matching of branches between frames. Currently, this is usually done manually [24–26]. However, manually matching branches throughout a video can take as long as initially tracing them, thus doubling the time requirement for direct user input. These movies can be hundreds of frames long, and each frame can contain many branches. Manual branch matching thus requires a lengthy human time commitment, and with high volumes of data there is a significant chance for human error. There is thus an immediate need for a way to automatically identify matched branches between frames.

Once matched branches have been identified, it is also desirable to have a statistically well-founded method to analyze the underlying patterns and changes over time. Patterns in these rapid morphological changes can yield deeper insights into the mechanisms behind the complex connective changes and adaptations. Statistical analysis can aid in determining patterns that may not be immediately or visually apparent through long time-series.

For testing connectivity theories, a seminal example of wiring is the development of the retinotectal connection. With its topographic organization and superficial location, the retinotectal system is a paradigm model for studying early neural wiring of patterned connectivity [31–37]. The zebrafish retinotectal system shares fundamental molecular pathways with other vertebrates (including mammals), and provides the additional qualities of being small, developing rapidly, and easy to image [38]. Retinal ganglion cells (RGCs) are the sole output cells of the eye. Their axons exit the eye, cross the midline, and the majority enter the contralateral optic tectum, where they seek out an appropriate termination zone to form a dense arbor of synapses. Branching of RGC axons on the optic tectum can be divided into two phases. The first phase, which we term pathfinding, occurs as axons extend dynamic branches as they travel caudally and search for an appropriate termination zone (TZ). The second phase occurs in early arborisation, as the axons cease to move forward through the tectal neuropil and instead focus on extending and retracting branches in a local area to develop appropriate synaptic connections. The branches that form this terminal arbor remain plastic [39, 40]. The plasticity initially sharpens the topographic map in response to visual input in a period with high levels of branch turnover [41]. In a similar manner to frogs and other fish, zebrafish retain connective plasticity throughout growth and adulthood, allowing newborn cells on the periphery of the retina to connect to appropriate locations on the topographic map in the tectum, which adds cells to the medial and caudal edges [42–44].

Here we address the issue of automatic branch matching by introducing Dynamic Time Warping (DTW) analysis as a method for automatically matching branches between frames. DTW is an algorithm that measures similarities between two sequences, which may occur over different time scales or speeds. DTW was originally a method to match patterns in time series data, however it has been applied to diverse two-dimensional spatial data, including fingerprint matching [45], walking patterns [46], speech recognition [47], and computerised handwriting recognition [48, 49]. Here we apply DTW to a novel situation; the changing branch structure of axons. DTW follows branches between frames where individual branches may elongate, retract, appear or disappear. This creates an automated method of identifying the branches that are the same in subsequent frames, and allows the collection of a large data set of branch dynamics.

Once branches are uniquely identified, we then apply a theory of birth-death processes to the changes in branch number over time. A birth-death process is a type of Markov chain, which describes a set of random variables in a countable state space that satisfies the Markov property: the probability of the next state depends only on its current state. A continuous-time Markov chain is where the time between jumps is continuous, defined as *t* in (0,∞) [50]. Thus, this type of stochastic model describes a random process where future transitions between different finite states over time are dependent only on the current state of the model. This type of model has previously been used to describe such diverse applications as population size [51], queueing theory [52], bacterial evolution [53], and epidemiology [54]. In our model, we define the state as the number of branches in the axon at each time point. Each branch of an axon is generated by a “birth” event, a branch can be removed by a “death” event, and in solving for the steady state we can determine the equilibrium number of branches produced by an axon. We can then look at how this state changes with time and behaviour. We investigate the rates of branch addition and deletion and their influence on the shape of the developing RGC axon over time in both normal conditions and after treatment with TTX to block neural activity.

We show that the birth rate changes over an axon’s life, especially between the pathfinding and arborisation phases of growth. The birth rate increases as axons reach a target zone, becoming higher as the axons build arbors. The death rate remains stable over the entire imaging period. Treatment with TTX causes a slight increase in the death rate of branches, but not the birth rate, providing a novel explanation for the differences found in a previous study [21]. Simulations driven by a constant death rate and an increasing birth rate generate numbers of branches through time that are consistent with the in vivo data. Thus, the transition between the pathfinding phase and arborisation phase of RGC axons is driven by a change in the birth rate of branches, which may be an important factor in determining axon morphology and connectivity in early brain development.

## Materials and Methods

### Ethics statement

All zebrafish procedures were performed with approval from The University of Queensland Animal Ethics Committee (approval SBMS/305/13/ARC). For more specific experimental details see [21].

### Biological data

We reanalysed a previously acquired data set [21]. Briefly, zebrafish were genetically labelled with a Brn3c:GAL4; UAS:mGFP transgene [55] resulting in sparse mGFP labelling of RGC axons. Half of the larvae were injected with tetrodotoxin (TTX), a paralytic toxin that disrupts neural activity. Pulled glass pipettes were used to inject 4.5–9 nL of 1 mM TTX into the yolk of anesthetized and immobilised zebrafish 1 to 6 hours before imaging. Fish with no spontaneous scooting behavior and that were non-responsive to touch were considered effectively paralyzed. The lack of neural activity was optically confirmed by confocal imaging of larvae carrying a genetically encoded calcium reporter gene in separate experiments [21]. Controls were injected with embryo media E3 (carrier alone). Zebrafish larvae were then immobilised in 1.5% agarose and positioned so that one tectum was roughly orthogonal to the imaging plane as in [23]. Confocal image stacks up to 60 *μ*m deep were taken through the optic tectum using a Zeiss LSM 510 inverted confocal microscope. The time-lapse movies covered up to 48 hours of zebrafish development between 2.5 and 4.5 days post fertilisation (dpf). Axons could begin growing into the imaged area on the optic tectum at any point after the recording started and were therefore temporally aligned using the time they reached their arborisation target zone/ termination zone (TZ) and ceased growing forward. Examples of these time-lapse movies have been included showing axon growth under control conditions (S1 Video) and after treatment with TTX (S2 Video).

The TZ position was defined by the convex hull linking the distal branch endpoints of the arbor in the final frame of each time-lapse movie. This arbor position was confirmed to be relatively stable when its position was compared between the final movie frame and images taken 24 hours later [23]. The time at which an axon reached the TZ was defined as the frame where the centroid of the convex hull first entered the TZ.

After imaging was complete, the larvae were removed from the agarose. Larvae which had been injected with TTX were monitored for movement. If any spontaneous or touch-responsive movement appeared, data from that larva were discarded. Typically, TTX-injected larvae regained spontaneous movements starting from 24 hours after the imaging ended [21].

Arbors on the tectum are mostly planar [25], and therefore time-lapse image stacks were flattened by maximum intensity projection. While arbors may have been curved along the dorsal surface, or been on slight angles from a true orthogonal image, these deflections were consistent between consecutive frames and would not alter branch matching or quantitative counts of branch numbers in each frame.

Ten control axons and ten TTX axons were traced using a custom built, semi-automated MatLab (Mathworks) program and the coordinates of each branch imported into Matlab.

### Dynamic Time Warping

Branches were traced manually. We then used Dynamic Time Warping (DTW) to identify branches that were maintained between two consecutive frames. We attached unique identifiers to the hundreds of branches that appeared and disappeared over 20–40 hours of growth for each axon. Tracings of two branches, *k* and *l*, in consecutive frames, *t* and *t* + 1, were defined as $B_{k,t}=\left\{b_{k,t}\left(x_{i},y_{i}\right),i=1,...,n\right\}$ and $B_{l,t+1}=\left\{b_{l,t+1}\left(x_{j},y_{j}\right),j=1,...,m\right\}$. Here *b*_*k*,*t*_(*x*_*i*_, *y*_*i*_) gives the *i*^*th*^ coordinates of the pixels in the branch *k* tracing at time *t*. The length of the two vectors *b*_*k*,*t*_ and *b*_*l*,*t*+1_ were *n* and *m* respectively.

A warping path *w* of the two branches was given by the set of *H* pairs of indices,

w=((i(1),j(1)),...,(i(H),j(H))). (1)

The warping path in Eq 1 describes *H* pairs of coordinates between branch *k* and branch *l*, such that all coordinates in branch *k* are matched to a coordinate in branch *l* at least once and vice versa. The warping path was determined such that the following cost function *C*[56] was small:

$$C\left(B_{k,t},B_{l,t+1},w\right)=\sum_{h=1}^{H}d\left(b_{k,t}\left(x_{i(h)},y_{i(h)}\right),b_{l,t+1}\left(x_{j(h)},y_{j(h)}\right)\right).$$ (2)

*H* was determined by the number of steps in the warping path. As the warping path must be non-decreasing [56], the branch tracings were oriented to be in the same direction (connected branch point to free end point) if they were not already. A warping path might be for example ((1,1) (1,2) (1,3), (2,4), … , (n,m)), so the first coordinate in branch k was matched with the first, second and third coordinate in branch l. A warping path therefore also met the boundary conditions of *i*(1) = *j*(1) = 1, *i*(*H*) = *n* and *j*(*H*) = *m*. The distance function used was the Euclidean distance between the two coordinates.

The overall DTW value was given by

$$D\left(B_{k,t},B_{l,t+1}\right)=\min_{w}C\left(B_{k,t},B_{l,t+1},w\right).$$ (3)

Eq (3) thus finds the minimum of the warping paths described by *C*. That is, the final DTW value *D* for each branch is equal to the minimum cost function (Eq (2)) that can be found in the subset of all possible warping paths for a branches in one frame matched against the selected branch in the previous frame.

The DTW value for all branch pairs between two frames was calculated, and then a match was made between two branches if the DTW value was a minimum for both branches. For example, frame *t* might have two branches *i* and *j* that could plausibly match to branch *k* in frame *t* + 1, such that the minimum DTW values for branches *i* and *j* are both with branch *k*. However, if the minimum DTW value for branch *k* is for branch *j*, then *k* and *j* will be matched, and branch *i* will be matched to its next closest branch or designated as being deleted between frames.

**Fig 1:**
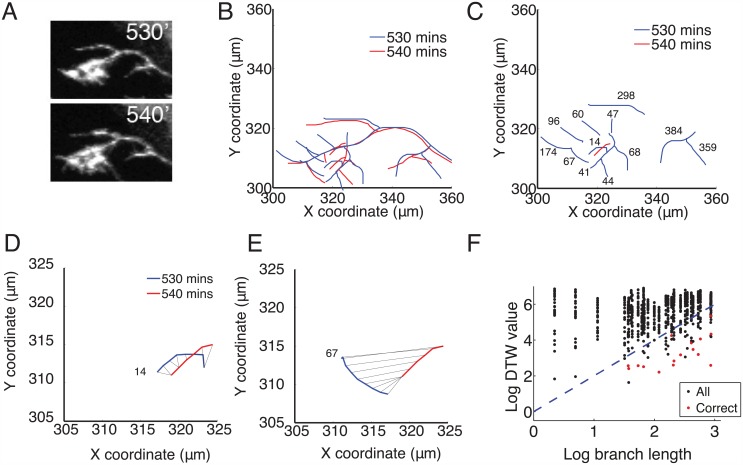
The Dynamic Time Warping (DTW) algorithm matched branches between frames based on location and length. (A): Stills froma time-lapse video at 530 and 540 minutes. Brn3C+ RGC axons were genetically sparsely labelled with mGFP. (B): Overlapped axon tracings at 530’ (blue) and 540’ (red). (C): Comparison of branches from 530’ (blue) to one branch from the 540’ frame (red). For clarity, the primary axon shafts are not shown. The number next to each blue branch is the DTW cost between it and the one red branch. Branches that are likely to be the same between two consecutive traced frames have the smallest DTW distance values. (D): DTW joins coordinates on the two branches (black lines) and assigns the lowest value (14) to the warping path, suggesting a correctly matched branch. This was confirmed by visual assessment of the tracings. (E): DTW comparison between the selected branch from 540’ and a branch further away gives a higher DTW value (67). (F): The log of the DTW distance between pairs of branches near the end of the time-lapse movie (1700’ and 1710’) versus the log of the length of the branches. The length of several branches could be similar, resulting in columns of matches that nearly overlap. The red points indicate correct matches as determined by visual inspection. The blue dotted line gives the maximum value for an acceptable branch match between consecutive frames, DTW = Length^2^, (the length of the branch in the first frame). This was included as a threshold for the automated matching of branches, and improved the reliability of the DTW algorithm. The branches with no corresponding red dot were correctly identified (compared to visual inspection) as having no match (deleted).

To confirm the accuracy of the DTW method, branch matching between randomly chosen pairs of consecutive frames was visually assessed. In the example described above, if branch *i* was sufficiently small then the minimum DTW value for branch *k* would be with branch *i* rather than branch *j*, even though it would be clear visually that branch *j* and *k* were more similar in length and position and more likely to be a correct match. To overcome this bias of shorter length branch selection, we included the heuristic threshold that a matched pair of branches must have a DTW value smaller than the squared length of the shortest branch in the pair (Fig 1F). This meant that shorter branches must be a closer ‘fit’ (that is, have a short warping path) to be matched compared to longer branches, which are more likely to have larger DTW values since they may retract part of their length or have slight changes in shape due to the original image quality.

### Interarrival time calculations

After each branch was uniquely identified, we modelled the stochastic processes of branch addition, deletion of branches and the time between two new branches appearing to determine how these parameters influenced the branch patterns of RGC axons during pathfinding and arborisation.

Branch statistics were modelled as a continuous-time Markov chain process using parameters for the forward transition probability *λ* and state-dependent backward transition probability, *μi*, where *μ* is a constant and *i* is the current number of branches on the neurite (not including the original axon shaft). This is equivalent to queuing theory models containing a multi-server queue with a constant probability of a new arrival, and any individuals in the system may be the next to exit (as opposed to a single-server queue, where only one selected individual may be the next to exit) [15]. For our system, the arrivals were the branch ‘births’, and any branch had an equal probability of ‘dying’, or being eliminated from the arbor. Therefore, the birth rate (*λ*) is proportional to the probability that a new branch will appear during each time step. The death rate (*μ*) is proportional to the probability of deletion for each individual branch at every time step, since every branch has a possibility of deletion at any given time. Thus, the overall rate of branch deletion for an axon is *μi*, which increases with the number of branches, *i*.

The steady-state solution of the number of branches on the neuron is given by a Poisson distribution with mean $\frac{\lambda }{\mu }$ [15]. This is derived using the Kolmogorov flow-balance equations, where the probability of the number of branches *j* once the stochastic process was in steady-state, is given by *π*_*j*_:

$$\left(\lambda +j\mu \right)\pi_{j}=\lambda \pi_{j-1}+\left(j+1\right)\mu \pi_{j+1},$$ (4)

$$\sum_{j=1}^{\infty }\pi_{j}=1.$$ (5)

Solving this gave the Poisson distribution,

$$\pi_{j}=\frac{e^{-\frac{\lambda }{\mu }}{\left(\frac{\lambda }{\mu }\right)}^{j}}{j!}\;j\geq 0.$$ (6)

Therefore, the number of axon branches has a Poisson distribution, which has an expected value of $\frac{\lambda }{\mu }$.

In order to estimate the birth rate (*λ*) and the death rate (*μ*) for each axon the branch lifetimes and interarrival times were calculated. An exponential distribution was fit to these two variables to find the parameters $\frac{1}{\lambda }$ and $\frac{1}{\mu }$. The exponential lifetime and interarrival times of our data indicated a Markovian process where the next state of the system depends only on its current state (that is, the current number of branches), since the exponential distribution is memoryless.

The recorded lifetime and interarrival times of branches were discrete, as they were captured at each movie frame taken ten minutes apart. Each branch birth or death could therefore have occurred at any time during the previous ten minutes. To account for this, we added (to the interarrival time following birth) or subtracted (from the lifetime following death) a random interval from zero to ten minutes for each branch.

### Continuous estimates of branch addition and deletion

To obtain estimates of the birth and death rates of an axon at different time points, branches were binned in ten-frame intervals (100 minutes). The initial interval was 0 to 100 minutes, then 10 to 110 minutes, and so on until the last frame. Similar estimates were obtained with an interval of six frames (1 hour), but we found that using a ten-frame interval resulted in a more robust measurement of averages. The birth and death rates were then estimated for the branches observed in these intervals, and branches still present at the end of the interval were treated as censored data.

Linear mixed effect models were fit using the lme4 package in R, with approximations of the significance of terms found using the lmerTest package [57]. Backward selection of variables was used, with higher order interaction terms removed first. Marginal and conditional R^2^ values were found using methods from [58].

### Simulations

Simulated axon branches over time were used to test the effects of different types of birth and death rates on the steady state (i.e., number of branches at any given time). The simulations were based on a birth-death process, where both the birth and death rates were exponentially distributed with various birth and death rates that were either constant, state-dependent or time-dependent. The time to the next event was given by log(*u*)/(λ* + μ*), where *u* ∼ *U*(0, 1) and λ*, μ* are different birth and death rates, respectively. The probability of this next event being a branch addition is given by λ*/(λ* + μ*).

## Results

### Dynamic Time Warping robustly identified branches through time

The branched structures of pathfinding and arborizing axons were first manually traced from a time-lapse confocal movie using a custom Matlab program and the coordinates and properties of each branch were recorded. We applied DTW to the resulting branch patterns (Fig 1). Briefly, DTW compared the similarities of two sequences (in this case, the branch coordinates) and found an optimal match between them by compressing or stretching aspects of one sequence to match the other. In our case, the warping and stretching allowed the unique identification of branches based on location and length, both of which could change due to branch elongation and retraction, movement in the imaging samples, and overall brain growth. DTW thus allowed a comparison of the structure of axon branches through time and uniquely identified the branches that remained present between movie frames, as well as those branches lost or gained. In a growing axon, branches can change substantially within 10 minutes (Fig 1A and 1B). Every branch in each frame was compared to the branches in the consecutive frame to determine whether the branch survived or was retracted between frames. When comparing a branch from a later frame (red line in Fig 1C) to the branches present at a previous time (blue) there may not be an exact overlap. DTW assigns each previously present branch a number *D* representing the difference between that branch and the branch from the next frame. Branches with low DTW values are considered matched (Fig 1D) while ones with high DTW values are considered different (Fig 1E). Examples of DTW values between one branch in one frame and all branches in the succeeding frame are shown in Fig 1C, and it is clear there is one best-matching branch.

One problem was that shorter branches could be biased towards being matched to incorrect branches in later frames, as they have lower DTW values in general. In principle, visual inspection can be used to rule out these incorrect matches. However, to automate the process, comparing log(DTW value) to 2log(branch length) provided a heuristic length threshold for automated branch matching (Fig 1F). For the 31 branches shown in Fig 1F, the smallest DTW value for any possible branch pairing (columns of black points) matched the branch chosen as a match by visual inspection (point shown in red). For several branches on the preceding frame, there was no correct match found (columns with no red points) indicating a branch that was retracted completely. This threshold was selected as it created an upper limit for DTW values that meant a relatively stricter criteria for a match between shorter branches compared to longer branches.

**Fig 2:**
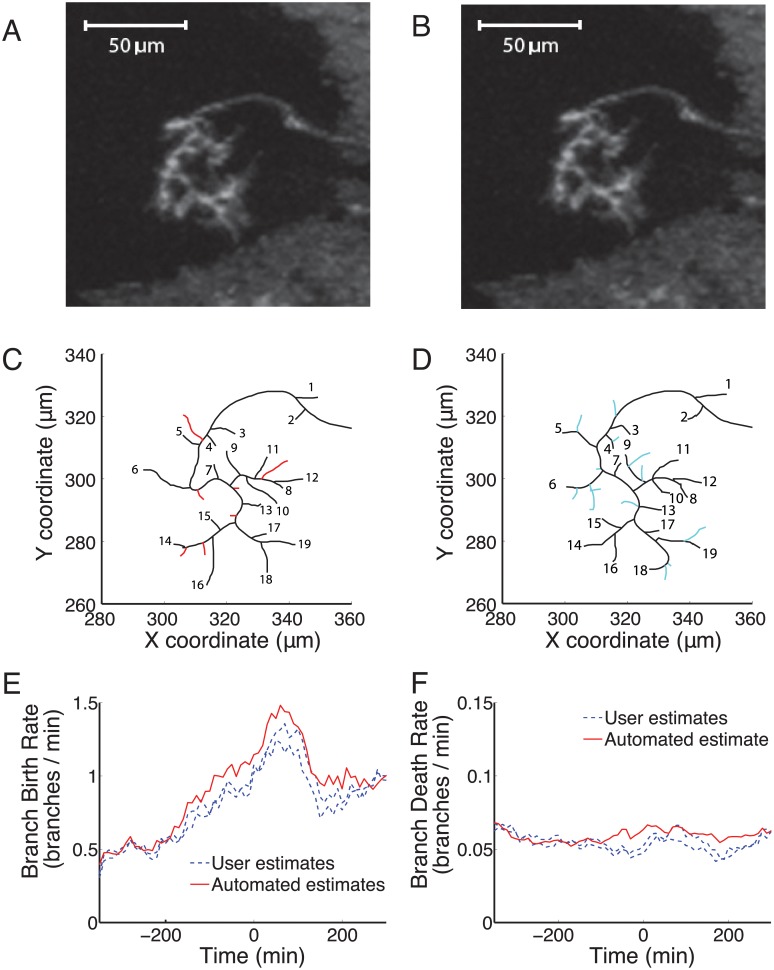
Branch matching using DTW identified branches that were removed, added or maintained between consecutive frames 10 minutes apart. (A) & (B): The original images of a control axon in two consecutive frames. (C): Seven branches (red) were removed completely or “died” between the two frames. (D): Thirteen new branches (cyan) were added or “born” in the ten minutes between the frames. Nineteen branches (numbered in both A and B) survived the interval (black), though they could have extended, retracted or shifted. (E): The branch birth rate for the DTW identified branches (automated, red line) was similar to that of two human users (dashed blue lines) who manually identified branches in one movie. (F): The death rate was comparable for branches either identified by DTW (automated, red line) or manually by two human users (dashed blue lines).

For each automated or visual comparison, two sequential frames were contrasted (Fig 2). DTW analysis successfully identified branches that were removed (Fig 2A) or added (Fig 2B) between frames. From this, lifetime data was gathered from the branches matched between frames. Each individual branch was assigned times for its birth and death, marking the appearance and disappearance of that particular branch, with the time between these events defining the branch lifetime. Interarrival time measured the elapsed time between branch ‘births’ on each axon arbor.

In order to test how reliably the automated DTW identified the same branch in different frames, we compared the DTW results to manual branch identification done by two individuals for one movie containing 88 time-lapse frames (14 hours 40 minutes of axon growth and branching). The branch birth and death rates over time produced by the DTW algorithm did not vary more than the two individuals matching branches by hand. The pattern of peaked branch birth rate and near constant death rate were maintained in all cases (Fig 2C and 2D). The total number of branches defined by each of the two users and the DTW algorithm differed only slightly (643, 673, or 727 unique branches). The DTW algorithm therefore did not depart markedly from the variation present between two human individuals.

The reliability of the DTW algorithm was also tested by randomly selecting traced frames from the 10 control axon movies, and then attempting to match branches between these pseudo-consecutive frames. The coordinates of the axon tracings were shifted so the traced frames had the same centre, and thus the two sets of branches close together, similarly to genuinely consecutive frames. The average matched number of branches over 230 random frames was 1.04 (±1.40 standard deviation). As a comparison, the control axon movies which had not been shuffled had an average of 11.44 matched branches over 230 frames (± 5.41 standard deviation). Thus, the rate of false matching between unrelated frames was very low.

### Birth and death rates predict the steady state distribution of branches

To model finite, countable states, such as the number of branches on an axon, a continuous-time Markov chain process can be used [15]. The expected number of branches on an axon can be predicted from the birth and death rate of the branches. The death rate of an axon’s branches is the inverse of the average lifetime of the branches, and is a measure of how long an individual branch is expected to survive. The birth rate is the inverse of the average interarrival time between new branches on the axon. Intuitively, higher birth rates indicate a higher number of added branches in a given interval of time.

**Fig 3:**
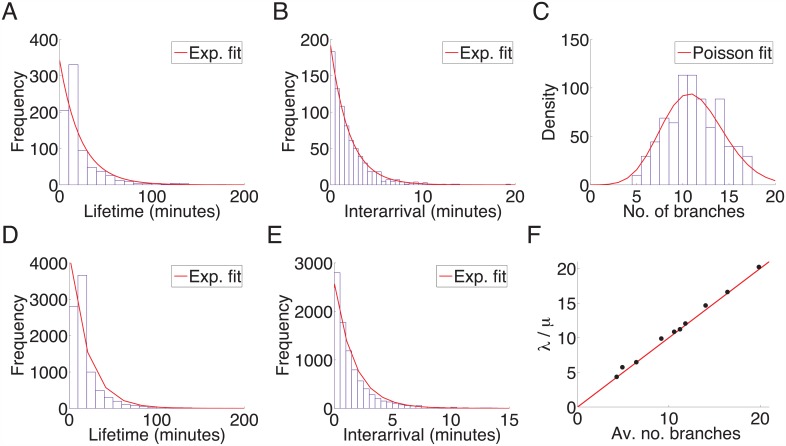
The lifetime and interarrival statistics from control axons fit an exponential distribution, and the number of branches on an axon is well represented by a stochastic birth-death process. (A): The histogram of the lifetime of the branches and the fitted exponential distribution with death rate ($\hat{\mu }=0.044$ (with a 90% *CI* of 0.041, 0.047)) from one axon with 787 branches. (B): The histogram of the interarrival time between branches and the fitted exponential distribution with birth rate ($\hat{\lambda }=0.49$ (0.46,0.52)) from the same axon. (C): The distribution of the number of branches in each frame (10 minutes apart) for one axon. The red line shows the fitted Poisson distribution with parameter 11.22 (10.70,11.74). (D): The lifetime distribution of branches from 10 control axons with 8876 total branches. The fitted exponential parameter for the death rate was $\hat{\mu }=0.048$ (0.047, 0.049). (E) The interarrival time distribution of branches from the 10 control axons. The fitted exponential parameter for the interarrival time was $\hat{\lambda }=0.5883$ (0.5761, 1.503). (F): The ratio of the lifetime and interarrival parameter estimates against the average number of branches for 10 axons. The red line shows $\hat{\lambda }/\hat{\mu }$ equal to the average number of branches, demonstrating that the average number of branches the axon has depends on the unique birth and death rates of its branches.

Initially, we characterised the distributions of the branch lifetimes, branch interarrival times, and number of branches for individual axons, as determined from the DTW analysis. For individual axons, both the lifetime and interarrival times were well fit by exponential distributions. Fig 3 shows the exponential distribution fit of branch lifetimes (Fig 3A), interarrival times (Fig 3B) and the Poisson distribution fit of the number of branches (Fig 3C) from one representative control axon (*n* = 787 branches total). The example axon had branches with a death rate of $\hat{\mu }=0.044\;\left(0.041,0.047\right)$ (where the numbers in brackets represent the 90% confidence interval) and a birth rate of $\hat{\lambda }=0.49\;\left(0.46,0.52\right)$ branches per minute. The ratio of the exponential parameters ($\frac{\hat{\lambda }}{\hat{\mu }}=11.22\;\left(10.70,11.74\right))$ was close to the mean value of the number of branches over frames. Thus, the distribution of the branch number over time matched the prediction from a birth-death process (see Materials and Methods).

In Fig 3D and 3E, all branches from the ten control axons were combined. The lifetimes and interarrival times of the grouped results were also well represented by an exponential distribution. Furthermore, for each of the ten control axons taken individually, the ratio of $\hat{\lambda }$ to $\hat{\mu }$ was close to the average number of branches for all axons (Fig 3F). This indicated that the fitted birth rate and death rate estimates were good indicators of the steady state distribution of branches.

However, a Poisson distribution was not a good fit to the mean number of branches (11.88) averaged across all grouped axons. The ratio of the parameter estimates from the grouped results was 12.22, greater than the mean number of branches. The grouped results therefore did not appear to follow the same trend as for individual axons (e.g. Fig 3C), where $\frac{\hat{\lambda }}{\hat{\mu }}$ was nearly the same as the mean number of branches per frame. This may be due to the large variation in total branch numbers between axons.

### Branch birth rates increased closer to the termination zone

So far we assumed that the birth and death rates were time homogeneous (constant over time), although the death rate of the axon overall was state dependent and proportional to the number of branches on the axon at any given time (*μi*, where *i* is the number of current branches). However, during the time that the RGC axons were imaged, branches were first extended when the axon was navigating towards a final termination zone (TZ) where axons stopped moving forward and established an initial arbor. The two growth phases observed in axons on the optic tectum (navigation and arborisation) have different morphological structures, with axons that have reached their TZ having larger numbers of branches and more elaborate arbors [23, 59–61].

**Fig 4:**
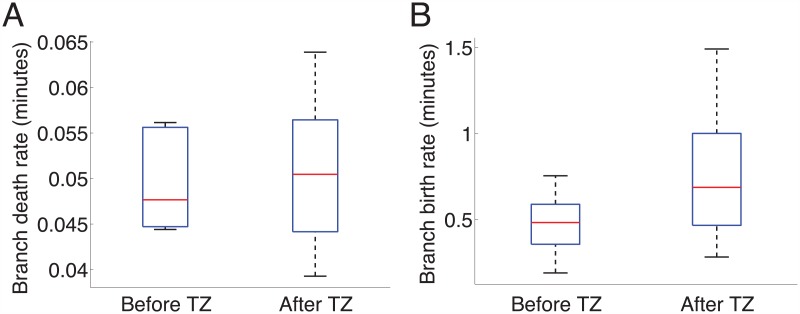
The birth rate of new branches increased after the axon reached the termination zone (TZ). The fitted exponential parameters of branches that appeared before or after the TZ was reached were compared for ten axons. (A): There was no difference detected in the estimated death rate, $\hat{\mu }$, of branches before or after the TZ was reached (p = 0.52, paired t-test). (B): There was a difference in the birth rate, $\hat{\lambda }$, of branches before and after the TZ was reached (p = 0.004, paired t-test).

We did not observe a significant difference in the death rates of branches before or after reaching the TZ (Fig 4A) (p = 0.5, paired t-test with *n* = 10; p = 0.3, Anderson-Darling (AD) test of normality). However, we found that the estimated birth rates for branches were significantly different when branches were categorized into those born before the TZ was reached, or afterwards (Fig 4B) (p = 0.004, paired t-test with *n* = 10; p = 0.2, AD test of normality). This indicated that the birth rates of an axon’s branches were dependent on the overall time that the axon had been growing, or alternatively, on its distance to the termination zone. Thus the differences in axon structure between the pathfinding and arborisation growth phases were due to changes in the rate of branch addition rather than the death rate.

**Fig 5:**
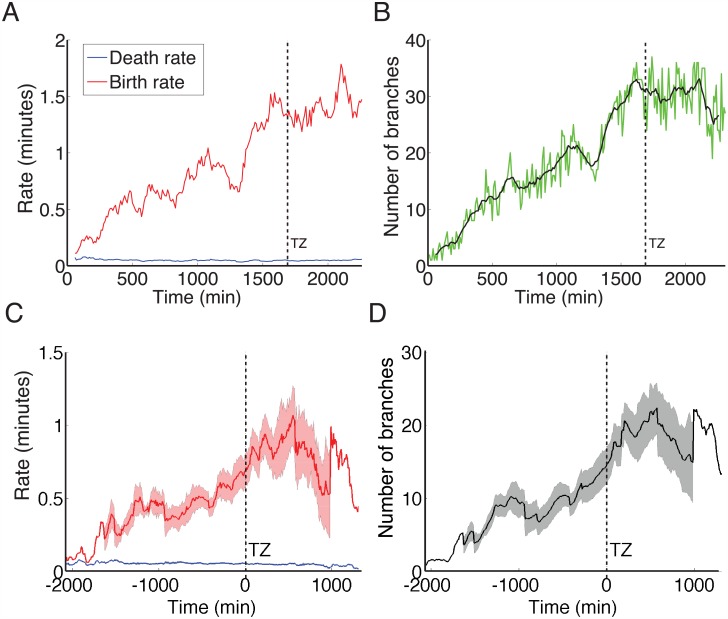
The branching rate estimates over time showed that the branch birth rate increased prior to the axon reaching the TZ. (A): A typical example control axon where the death rate (blue) was relatively constant compared to the birth rate (red). There was an increase in the branch birth rate before the axon reached the TZ, at which point the rate became more constant. (B): The number of branches (green) of the axon in (A), and the moving average of the branch number (black) with a window of 100 minutes. The number of branches followed a similar pattern to the birth rate. (C): The averaged rates over all control axons, after times were aligned such that zero was the point the TZ was reached. Mean branch birth rate (red) with shaded SEM showed that the birth rate peaked after the TZ was reached. The average death rate (blue) stayed relatively constant, with a SEM too small to be visible. (D): The average number of branches for all axons over time, with shaded SEM, reflected the increase in the birth rate of branches.

Next, we investigated how branch birth and death rates changed over time continuously, regardless of the axon’s distance to the TZ. In order to establish a continuous estimate of the branch birth and death rates, branches were grouped within windows of ten time-lapse frames (each spanning 100 min) and exponential distributions were fit to these 100 min subsets in order to investigate whether these rates varied between subsets, i.e. were time inhomogeneous. This revealed that the birth rate of the axon branches increased with time, as the axon approached the termination zone. About 100 min before the termination zone was reached, the branch birth rate increased to greater than one branch per minute (Fig 5A). The death rate of branches from this representative axon stayed relatively constant over time.

The numbers of branches counted in each frame over time (Fig 5B) followed a pattern similar to the branch birth rate (Fig 5A). This again indicated that the total number of branches was controlled by a change in the birth rates of branches rather than the death rate. At periods of low branch birth rate, such as around 1250 minutes in the example axon, the total numbers of branches also decreased.

When averaged, the combined results of all control axons showed an increasing birth rate until 500 minutes after the termination zone was reached, and then a gradual decline (Fig 5C). The averaged death rates declined slightly, but, although significant, this was small compared to the change in the averaged birth rate. For the grouped axons, the average number of branches over time also followed a similar trend to the birth rate (Fig 5C and 5D).

As the birth rates of branches were not constant over time, a time-homogeneous Poisson process was not adequate to describe the number of branches in these axons. We therefore used a time-inhomogeneous Poisson process, where the birth of new branches was more likely when the axon is closer to the TZ. The probability of a branch deletion changed very little over the same time frame.

### TTX increased the death rate of axon branches

**Fig 6:**
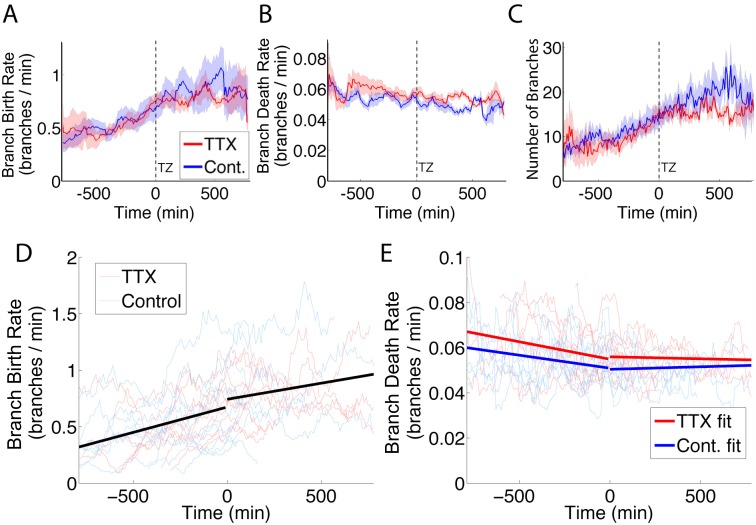
TTX treatment increased the death rate of branches but did not alter the birth rate. (A): The averaged (± SEM) branch birth rate for TTX-treated (red) and control (blue) axons increased until the TZ was reached and then plateaued. Times were aligned to the point the TZ was reached. (B): The averaged branch death rate (± SEM) was slightly higher for TTX-treated conditions (red) than for controls (blue), particularly after the TZ was reached. (C): The number of branches were averaged for each group over time (± SEM) and axons under control conditions had a higher number of branches than axons in TTX-treated environments after the TZ was reached. (D): The fitted model (including fixed effects only) of the birth rates (black). The slope and intercept of the birth rate changed after the TZ was reached. The individual axon results are shown for controls (blue lines) and axons in TTX-treated environments (red) in the background. (E): The fitted model for the average death rates of the TTX axons (bold red) was higher than that of controls (bold blue), shown over a background of the raw traces (thin lines). The fitted intercept for the TTX branches was higher than the control branches. See Table 1 for statistical tests relating to this figure.

In control conditions, a time-dependent birth rate and a constant but state-dependent death rate described the number of branches over an axon’s navigation and arborisation (Fig 5). The experimental data also included a group of ten axons that navigated and arborised while neural activity was abolished with tetrodotoxin (TTX), originally presented in [21]. We investigated whether the loss of neural activity affected the birth and death rates of branches. Similar to controls, these axons were analyzed using DTW to determine branch lifetimes and death rates. The ten axons treated with TTX displayed similar patterns in continuous birth rate estimates as the control axons (Fig 6A). However, the death rates were higher in the TTX axons than the controls (Fig 6B). The average number of branches in the TTX axons also decreased more than that of control axons after the TZ was reached (Fig 6C), consistent with the higher death rate of the TTX branches. Thus a decreased branch lifetime was responsible for the lower numbers of branches in TTX treated conditions.

Linear mixed effect models were fit to both the $\hat{\lambda }$ and $\hat{\mu }$ estimates in order to treat time as a continuous variable and include the substantial variation between the axons. (e.g. individual variation shown in Fig 6D and 6E). The fitted models are shown in Table 1. Individual axons were chosen to be a random effect on both the intercept (the starting estimated rate) and the slope dependent on time, due to the observed variance of the slope and intercepts between axons. The slope and intercept will always be different between different sampled axons from the entire population. However, despite this random starting point, the response (number of branches) for each individual sample axon did change consistently with time. The estimated fixed effects of time and the intercept on the model are shown in Fig 6D.

**Table 1:** Linear mixed effect models were fit to *μ* and *λ* estimates using backward stepwise variable selection, with random intercept and slope terms due to the variance between individual axons. The table shows the coefficient, the standard error and the p-value, with degrees of freedom estimated by Satterthwaite approximations. $\hat{\mu }$ does change between control and TTX axons, while $\hat{\lambda }$ does not. Both $\hat{\lambda }$ and $\hat{\mu }$ have a strong interaction between time and whether the axon has reached the TZ or not. Models were only fit to time points when there were at least two TTX and two control axons (-13 hours to +13 hours). Marginal *R*^2^ for $\hat{\mu }$ is 0.11 (considering fixed effects only), conditional *R*^2^ is 0.48 (including random effects). Marginal *R*^2^ for $\hat{\lambda }$ is 0.27, conditional *R*^2^ is 0.66.

Rate	Fixed effects					Random effects (variance)	
	Time	TZ	Type	TZ*Time	Time*Type	Intercept	Time
$\hat{\mu }$	1.3 × 10^−6^	−3.0 × 10^−4^	4.7 × 10^−3^	−1.4 × 10^−5^	−2.2 × 10^−6^	3.4 × 10^−5^	4.4 × 10^−11^
	2.7 × 10^−6^(0.64)	6.3 × 10^−4^(0.63)	4.1 × 10^−4^(< 0.001)	1.8 × 10^−6^(< 0.001)	1.2 × 10^−6^(0.053)		
$\hat{\lambda }$	2.8 × 10^−4^	−6.9 × 10^−2^		1.6 × 10^−4^		3.3 × 10^−2^	9.7 × 10^−8^
	1.1 × 10^−4^(0.021)	1.6 × 10^−2^(< 0.001)		4.5 × 10^−5^(< 0.001)			

The best model for the estimated birth rate, $\hat{\lambda }$, was not dependent on the treatment condition of the axon. It did, however, determine that whether the axon had reached the TZ or not did influence the branch birth rate. Both the intercept term for the TZ (*p* < 0.001) and its interaction with time (*p* < 0.001) were significantly different from zero, and thus included in the model. This confirmed the pattern that was observed in the mean results across axons seen in Fig 5. There was an increase in the birth rate of branches as the axon reached the TZ, and a decline in that increase over time. This was unchanged with TTX treatment.

For the selected model of estimated branch death rate, $\hat{\mu }$, the treatment condition of the axons was significant (*p* < 0.001). The death rates for the TTX axons were higher than the control (0.055 per minute for TTX axons versus 0.050 per minute for control axons at the time the TZ is reached). The interaction effect of time and the axons arrival at the TZ was also significant (*p* < 0.001), such that the death rate was slightly higher for axons before the TZ was reached. Thus, branches were less likely to disappear after the TZ was reached for both treatment groups, and the TTX axon branches died at a slightly higher rate than the control branches.

### Simulations of inhomogenous birth-death processes reproduced experimental results

Under control conditions, the state of the axon, i.e. the number of branches, increased with time, before reaching a plateau. Several explanations could account for the observed increase. To conceptualise the possible differences in the birth and death rates of the axons, and to confirm the observations on the in vivo axons, birth-death processes were simulated using biologically plausible rates taken from observations on the in vivo axons. We investigated three possible models.

- Model 1 had a constant branch birth rate and a state-dependent death rate, which increased along with the number of branches.
- Model 2 had a death rate that was both time-dependent and state-dependent.
- Model 3 had a state-dependent death rate and time-dependent birth rate.

All three models produced similar steady state results (the average number of branches once the axon reached the TZ), but there were differences in the patterns of branch numbers prior to reaching the TZ, which suggested that the three models differed in their ability to replicate pathfinding related branching during axon navigation.

**Fig 7:**
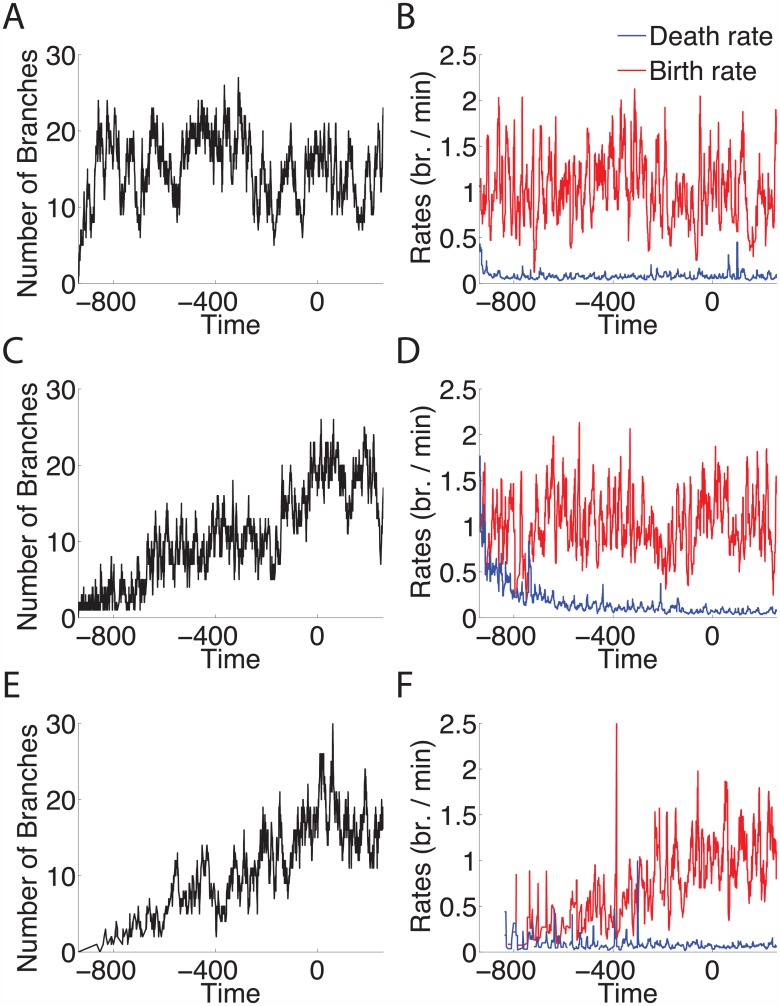
Simulations of simple birth-death processes showed that a changing birth rate and constant death rate are most consistent with the data. Each simulation had a forward transition rate of *λ*, and backward transition rate of *μi* (where *i* is the number of branches). Time axis is relative to arrival at the TZ. **Model 1:**(A): Number of branches over time with constant arrival rate of 1 branch per minute, and constant death rate of 0.06 branches per minute. The number of branches reached steady state with a mean of 1/0.06 = 16.67 branches. (B): The estimated birth rate (red) and death rate (blue) for the simulated number of branches in Model 1. **Model 2:** (C): Number of branches over time with constant birth rate of 1 branch per minute, and death rate of 1/(0.0167*t*) for *t* < TZ and 0.06 for *t* ≥ TZ. (D): The estimated rates for the simulated branches in Model 2. **Model 3:** (E): Number of branches over time with constant death rate of 0.06 branches per minute, and birth rate of 0.001*t* for *t* < TZ and 1 for *t* ≥ TZ. (F): The estimated rates for the simulated branches in Model 3. This simulation best reflects the observations from the experimental branch data.

For the Model 1 simulation (Fig 7A and 7B) the birth rate, *λ*, and probability of a branch death, *μ*, were constant over time. The steady-state solution to this process was a Poisson distribution with a mean *λ*/*μ* of 1/0.06 = 16.67. These rates were not time dependent and thus Model 1 represents a time-homogeneous process. Branch number over time did not increase; it reached a quasi-steady state far from the TZ, soon after the simulation was started (Fig 7A). This model suggested that the time-dependency of the experimental data was a necessary component.

One way to gain an increasing number of branches over time is through a time-dependent decrease in the death rate, coupled to a constant birth rate. This is demonstrated by the simulation of Model 2 (Fig 7C and 7D). In this model the death rate initially decreased with time before becoming constant after the axon’s arrival at the TZ. Simulating the branching birth and death rates in this manner allowed for number of branches to be similar to the experimentally observed measurements of axons.

However, our data suggested that the death rate of branches stayed relatively constant while the birth rate increased closer to the TZ. This is reproduced by the simulations for Model 3 (Fig 7E and 7F). The branch number over time for the simulations was similar to the measures of control axons (e.g. Fig 3C).

Thus, the simplest description of axon branching matching the data was an inhomogenous birth-death process with forward transition rate of adding branches *λ*(*t*) and backward transition rate of deleting branches *μi* (where *i* is the current number of branches). The likelihood of a branch addition increases with time (that is, as the axon moves closer to the TZ), and the likelihood of the deletion of any single branch remains relatively constant.

## Discussion

Axon branching is an essential step in the establishment of neural circuits [62]. In this study, we used quantitative methods to extract new information about the nature of dynamic branch behaviours. We introduced Dynamic Time Warping as a method to automatically match branches between frames. We also modelled the branch dynamics as a birth-death process, i.e. a special case of a continuous-time Markov process. From this model, we determined that the birth rate of branches of zebrafish retinotectal axons increased over time. Under control conditions, the death rate remained constant. After activity was blocked with TTX, the death rate of branches increased slightly, however the birth rate was unchanged. Additionally, we extracted average birth and death rates from the data and used them to parameterize computational simulations of branch dynamics, and the statistics of the simulations closely matched the statistics of the biological data. Together these results revealed underlying features of the biology of retinotectal pathfinding and arborisation.

### Branching and molecular guidance cues

What biological factors could underlie the patterns we observed? A variety of guidance cues present on the optic tectum are known to affect both branching and axon guidance. The increasing branch birth rates and subsequent rate plateau as the axon arrives at the TZ (and therefore stops moving through the gradients) could be caused by the growing axon encountering changing levels of Ephrins [63, 64], Semaphorins, [65–67], Netrin-1 [68, 69], or Slit proteins [62, 70–73] found on the optic tectum. Additionally, the presence of target derived growth factors could encourage the increasing branch birth rates, including tectal sources of fibroblast growth factor (FGF) [74, 75], nerve growth factor (NGF) [76], and brain derived neurotrophic factor (BDNF) [77–80]. These extrinsic cues may act exclusively on the branch birth rates, while the unchanging death rate may be derived from a more intrinsic process. Retinal axons have previously been shown to respond to topographic cues even in the presence of TTX [81]. Our similarities in the branch birth rate with and without TTX show independence from activity in a similar manner.

### Branching and neural activity

Besides molecular cues, neural activity also plays an important role in the development of connectivity. For retinal ganglion arbors, neural activity sculpts the final morphology [25, 41, 82–85]. Transient branches become more stable in the presence of their synaptic targets [86, 87], and the maturation of synapses stabilizes branches [41, 88]. Therefore, the higher death rate that we observed when using TTX may be due to interference with target identification and synaptic maturation caused by the lack of activity.

If depolarisation is suppressed, axons may have fewer branches and maintain immature morphologies [89]. Previously, we likewise found fewer numbers of branches after treatment with TTX [21] and here we show that this is due to the higher probability of branch death when neural activity is blocked. Over time, this increase in death rate may prevent the more complicated mature morphologies from forming. It is possible that the lower numbers of branches seen in mammalian cortical slice cultures after neuronal silencing could also represent a lower proportion of surviving branches rather than a lower number of branches initiated [90, 91].

Our current work did not address correlated activity, as neural activity was globally silenced, however, under control conditions it is likely that correlations between pre- and post- synaptic activity also play an important role. When correlated activity is detected pre- and post-synaptically, NMDA receptors and retrograde messengers stabilize RGC axon branches and suppress new additions and deletions [92–94]. Thus, the lack of global activity in our experiments leads to the loss of correlated activity and may therefore increase the death rate through an active deletion process for mismatched branches.

### Biological significance

The increase in the death rate with the application of TTX is small. This agrees with previous assessments where TTX had limited effects on the morphologies of pathfinding axons, which showed small but significant decreases in branch numbers, length and area covered at certain times during pathfinding and early arborization. [21]. However, an important caveat is that our imaging conditions may have reduced the effects of blocking activity. The imaging took place inside a darkened room, where visual input was extremely limited. Under natural conditions, zebrafish receive visual input during this time period, as functional connections develop by 72 hpf and our imaging analysed larvae from approximately 60 to 100 hpf [21, 95–97]. The restoration of natural visual input during this time might therefore increase the effect of blocking activity. In control cases, if wiring followed Hebbian learning rules and stabilized the correlated connections, the difference when TTX increased the death rate would likely result in more profound morphological changes. Without visual input as a positive force driving stabilization of the ‘proper’ connections, the resulting differences in dynamics between control and TTX treated larvae may remain more subtle.

### Extensions for DTW and Markov chain modeling

DTW represents a novel way to extract information about movement and growth of living tissue when imaging for long periods in vivo. DTW will likely be useful for systems where the neurons have large arbors or are imaged for long periods of time, and could also be used to analyze older data sets to extract new information. Several recent studies have examined the alterations in dendritic spines during learning, navigation and memory [98, 99]. DTW could be expanded to include spine analysis, and contribute to studies on the growth and stabilization of dendritic spines during learning. It could also be useful for examining changes in structure, which along with activity patterns, may underlie behaviors including decision making, learning, and memory [100], as well as experience dependent structural reorganization [101].

Regarding the reliability of DTW, we found that comparing random frames of axons produced a very low branch matching rate, and that the DTW method was correct in labelling the majority of the branches as ‘deleted’ in each frame. Measuring a rate of true positive branch matching is difficult, since there is no ‘ground truth’ of correct branches due to the variance in the axon morphologies. The DTW estimates for the branch birth and death rates were slightly higher than both manual users (Fig 2E and 2F), suggesting the possibility of a consistent failure to match branches across time points. However, we have insufficient data to conclude that the DTW results were outside the range of human variability.

Even the manual users were somewhat inconsistent. The continuously estimated birth and death rates for the branches matched by the two manual users were slightly smaller than the estimated birth and death rates for the DTW matched branches (from Fig 2E and 2F). This suggests that there was a consistent failure rate at matching branches across time points, assuming that the DTW results are outside the range of human variability.

We found that our axon branches followed a continuous-time Markov chain model, specifically a birth-death process model. This model allowed us to alter experimentally derived parameters to determine the changes in the output state (the numbers of branches at each timepoint). We found that there were two different solutions, depending on whether the axons were pathfinding or had reached their TZ. Applying this type of model to other neurons and different experimental situations will allow a richer quantitative analysis. In particular, similarities and differences across different neurons can be compared to yield insights into the mechanisms underlying neuronal guidance, branching, and connectivity.

### Conclusions

We have applied DTW to a novel field and found it to be a powerful tool in matching complex dynamic structures against a growing and shifting in vivo environment. This tool will be of benefit to studies on the development of neurons over time. Additionally, we showed that the addition and deletion of RGC branches could be well matched by a continuous-time Markov chain process, and that this model could separate changing phases of growth over a neuron’s development. These models could potentially be applied to other brain areas. Indeed, it will be intriguing to characterise the progression of branching in other brain regions, to determine the interplay between general rules for growth over time and individual, intrinsic axon programming.

## Supporting Information

S1 Video RGC axons growing into the optic tectum in control conditions. Compressed video representing 42 hours of development in 252 time-lapse frames starting around 2.5 dpf. Confocal stacks are represented with maximum intensity projections. RGC axons were genetically labelled with a Brn3c:GAL4; UAS:mGFP transgene. Oblique dorsal view focused on the optic tectum, anterior to the left. File: control.mp4
(MP4)

S2 Video RGC axons growing into the optic tectum in a zebrafish larva injected with TTX. TTX was injected into the yolk sac at 2 dpf. Compressed video showing 41 hours and 40 minutes of development in 250 time-lapse frames starting around 2.5 dpf. Confocal stacks are represented with maximum intensity projections. RGC axons were genetically labelled with a Brn3c:GAL4; UAS:mGFP transgene. Oblique dorsal view focused on the optic tectum, anterior to the left. File: ttx.mp4
(MP4)
